# Always connected, not always balanced: the double-edged effects of after-hours work connectivity behavior on work–family conflict among civil servants

**DOI:** 10.3389/fpsyg.2026.1840536

**Published:** 2026-09-28

**Authors:** Bo Bi, Qing Liu

**Affiliations:** 1 Xuzhou Central Hospital, Xuzhou, China; 2 The Affiliated Xuzhou Municipal Hospital of Xuzhou Medical University, Xuzhou, China

**Keywords:** public service motivation, work connectivity behavior after-hours, work goal progress, work–family conflict, workplace anxiety

## Abstract

**Introduction:**

Drawing on the Work–Home Resources Model, this study examines the double-edged effects of work connectivity behavior after-hours (WCBA) on work–family conflict (WFC) among Chinese civil servants.

**Methods:**

We conducted a three-wave survey of 240 civil servants. The proposed parallel mediation and moderated mediation relationships were tested using regression-based analyses with bootstrapping.

**Results:**

WCBA indirectly reduced WFC by facilitating work goal progress, while simultaneously increasing WFC by heightening workplace anxiety. Public service motivation moderated both pathways. Higher public service motivation weakened the positive relationship between WCBA and work goal progress but strengthened the positive relationship between WCBA and workplace anxiety.

**Discussion:**

The findings reveal that WCBA can function as both a resource enabler and a stressor in public organizations. They extend the Work–Home Resources Model by identifying distinct resource-gain and resource-loss pathways and demonstrate that public service motivation may generate both beneficial and adverse consequences in an always-connected work environment.

## Introduction

The rapid development of information and communications technology (ICT), alongside ongoing government streamlining reforms, has profoundly transformed work practices in the public sector (Dawes, 2008; Roman et al., 2019). To meet increasingly complex service demands, civil servants are frequently required to perform work-related tasks during non-working hours using mobile devices and digital tools. In particular, in pursuit of a responsive, service-oriented government, many Chinese government departments have recently begun expecting employees to remain connected around the clock. For example, civil servants in Shanghai frequently monitor work-related information and citizens’ requests, responding promptly even after official working hours and on weekends (Wang and Li, 2023). This behavior-referred to as work connectivity behavior after-hours (WCBA; Ren et al., 2023; Schlosser, 2002)-has attracted substantial research attention. Studies have shown that WCBA can affect a wide range of work-related and non-work outcomes, including work performance (Ren et al., 2023), job satisfaction (Diaz et al., 2012), work engagement (Lanaj et al., 2014), organizational commitment (Ferguson et al., 2016).

Regrettably, despite numerous studies on the impact of WCBA on employees, research focusing on its influence on the non-work domains of Chinese civil servants remains limited. Although pioneering studies have examined the effect of WCBA on employees’ family lives, existing evidence continues to be inconsistent and inconclusive—particularly regarding work–family conflict (WFC), which French et al. (2018) define as the employees’ perception of inter-role conflict arising from competing demands in work and family domains. While some studies suggest that WCBA exacerbates WFC (e.g., Butts et al., 2015; Ferguson et al., 2016; Gadeyne et al., 2023), others report no significant relationship between the two (e.g., Golden, 2012; van Zoonen et al., 2020). Thus, the underlying mechanisms and boundary conditions of this influence still require further investigation. Furthermore, most existing research has focused on employees in corporate settings, while civil servants in modern public organizations have received considerably less attention (Macijauskienė and Stankevičiūtė, 2022; Liu and Shi, 2024; Wang and Li, 2023). With the rapid advancement of information technology and the expansion of e-government, few scholars have begun calling for more thorough investigation into how WCBA affects various aspects of civil servants’ personal and family lives (Liu and Shi, 2024; Wang and Li, 2023).

To address these gaps in the research, this study aims to investigate the dual-edged impact of WCBA on WFC among Chinese civil servants. We draw on the Work-Home Resources (W-HR) Model as the overarching framework for developing our hypotheses. The W-HR Model links demands and resources in one domain to outcomes in another through personal resources, thereby providing a systematic explanation of the causal mechanisms and boundary conditions underlying work-family relations (Ten Brummelhuis and Bakker, 2012). Specifically, we explore the potential parallel mediating pathways (resource-gain path vs. resource-loss path) through which WCBA may simultaneously increase and decrease WFC. On one hand, WCBA enables employees to achieve greater work goal progress, which strengthens their sense of accomplishment and indirectly alleviates WFC. This represents the resource-gain path. On the other hand, WCBA also heightens workplace anxiety by extending work demands beyond normal hours, thereby depleting personal resources and exacerbating WFC. This represents the resource-loss path.

Additionally, the W-HR Model emphasizes that personal resources reflect how individuals cope with stressors and mobilize other resources. Prior empirical research has identified and confirmed the moderating role of resource factors such as intrinsic motivation and prosocial motivation in linking work resources with personal resources (e.g., Bakker et al., 2019; Ilies et al., 2017; Lin et al., 2017). Therefore, this study considers the moderating influence of Public Service Motivation (PSM), a distinctive attribute of public employees, in shaping these complex relationships. PSM refers to an individual’s predisposition to respond to motives grounded in public institutions and organizations, including the desire to serve the public interest and contribute to society (Perry, 1996). When engaging in WCBA, public employees with high PSM often regard public service and altruism as a mission, which may lead them to overlook personal work goals and thereby weaken the positive effect of WCBA on goal progress. At the same time, their strong sense of duty and internalized pressure significantly amplify workplace anxiety. In contrast, employees with low PSM tend to prioritize self-interest and are less driven by public values, making them more focused on concrete task goals and less likely to experience anxiety from WCBA. Taken together, the proposed research model is depicted in Figure 1.

**Figure 1:**
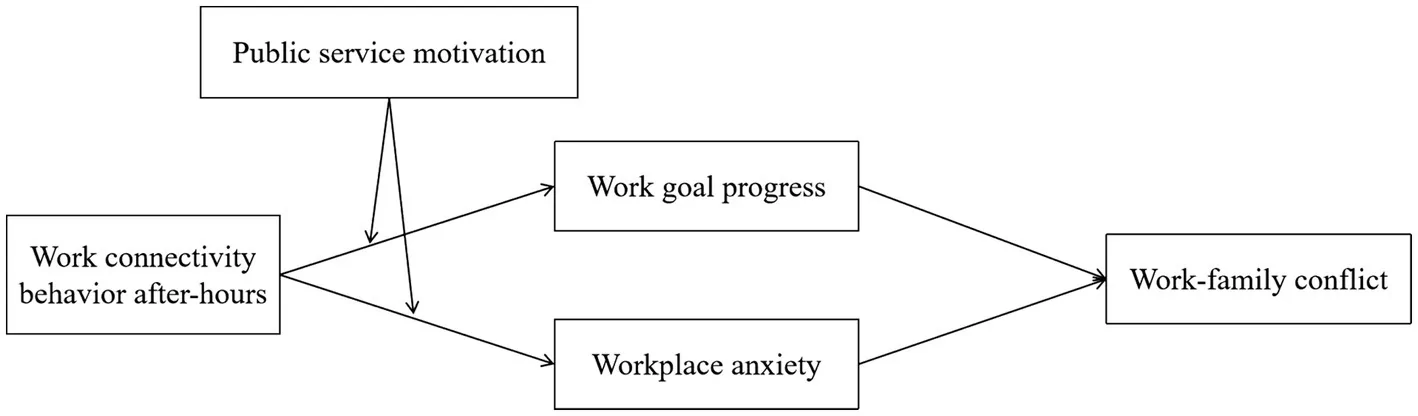
Hypothesized model.

## Theory and hypothesis

### Work connectivity behavior after-hours

WCBA, first introduced by Richardson and Benbunan-Fich (2011), refers to employees’ engagement in job-related tasks or work-related communication outside formal working hours through portable information and communication technologies. Although prior studies differ in their precise definitions (e.g., Derks et al., 2016; Dettmers et al., 2016; Fenner and Renn, 2010), WCBA is generally characterized as work activities performed beyond standard working hours (e.g., after work or during holidays) and outside conventional workplace settings (e.g., at home), facilitated by devices such as smartphones or laptops (Eichberger and Zacher, 2021). Measurement approaches in existing research typically focus on three dimensions: frequency (Fenner and Renn, 2010), duration (Wright et al., 2014), and intensity (Derks et al., 2016). With the widespread adoption of digital technologies and social media, WCBA has become an increasingly prominent topic in organizational and public management research. Empirical evidence indicates that WCBA is significantly associated with various work outcomes, including task performance, job engagement, job satisfaction, organizational commitment, and turnover intention (e.g., Ferguson et al., 2016; Ren et al., 2023).

Beyond its effects on formal work outcomes, WCBA also blurs the boundaries between work and family, requiring employees to manage job demands within personal or family contexts. As a result, scholars have increasingly examined its potential spillover effects on WFC. However, prior findings remain mixed: some studies suggest that WCBA exacerbates WFC (e.g., Butts et al., 2015; Dettmers, 2017; Ferguson et al., 2016), whereas others report non-significant associations (e.g., Golden, 2012; van Zoonen et al., 2020). These divergent results suggest that WCBA may simultaneously produce both positive and negative effects, highlighting the need to investigate the underlying mechanisms and boundary conditions that shape its impact.

In the Chinese context, the WCBA behavior of civil servants carries unique cultural and institutional significance. In the rapidly evolving public sector in China, there is a growing expectation for civil servants to remain connected beyond formal working hours, making the handling of work-related tasks after hours increasingly routine (Wang and Li, 2023). Traditional Confucian and Daoist values emphasize humility, altruism, and collective well-being, aligning closely with the public service ethos of “serving the people” (为人民服务, wèi rén mín fú wù), which has been long institutionalized in government systems. Civil servants are often expected to prioritize public interests over personal needs, and such dedication is culturally and institutionally encouraged (Chen et al., 2025). Yet, these expectations may also increase the likelihood that civil servants sacrifice personal and family well-being when engaging in WCBA. Despite its relevance, systematic research examining the effects of WCBA on non-work domains—particularly family life—among Chinese civil servants remains scarce, warranting further empirical investigation.

### Resource-gain path: the mediating role of work goal progress between WCBA and WFC

According to the W-HR Model, personal resources serve as a bridge between job demands, job resources, and outcomes in the family domain (Ten Brummelhuis and Bakker, 2012). When work resources enhance employees’ personal resources, they can generate positive outcomes in the family sphere, thereby alleviating WFC. In this study, work goal progress is adopted as the key indicator of this “resource-gain” pathway, which refers to employees’ evaluation of the extent to which their current performance approaches the desired goal level (Wanberg et al., 2010).

Engaging in WCBA can facilitate work goal progress by enabling employees to flexibly allocate time and effort toward work tasks and thus improve performance (Richardson and Thompson, 2012). WCBA facilitates employees’ access to additional job resources—such as timely information, task-related support, enhanced work flexibility, cross-domain resource flows, and strengthened communication with supervisors and colleagues (Fujimoto et al., 2016; Mazmanian et al., 2013; Duxbury et al., 2014). Job resources, in turn, provide instrumental and motivational support that help employees accomplish work goals (Demerouti et al., 2001). Hence, WCBA may promote employees’ work goal progress through the acquisition of valuable resources.

Moreover, work goal progress can help reduce WFC through two mechanisms. First, when employees experience higher work goal progress, they perceive themselves as closer to achieving desired goals, which increases satisfaction and a sense of accomplishment—emotional resources that spill over positively into the family domain (Lapierre et al., 2018). Second, achieving work goal progress facilitates employees’ personal growth and strengthens their capacity to fulfill family responsibilities, such as engaging in household decision-making, thereby reducing conflict between the two domains. Consistent with this logic, Tennakoon (2021) argued that WCBA enhances work flexibility, reduces stress, and fosters satisfaction and accomplishment, ultimately contributing to work–family enrichment and balance. Combining these arguments, we postulate:

*Hypothesis 1*: Work goal progress mediates the relationship between WCBA and WFC.

### Resource-loss path: the mediating role of workplace anxiety between WCBA and WFC

According to the Work–Home Resources (W-HR) Model, job demands can deplete employees’ personal resources, such as time, emotional energy, and cognitive capacity, which in turn limits their ability to fulfill family role requirements and exacerbates work–family conflict (Ten Brummelhuis and Bakker, 2012). In this study, workplace anxiety is adopted to represent this “resource-loss” pathway. Workplace anxiety refers to feelings of nervousness and apprehension about accomplishing job tasks (Eysenck et al., 2007; Muschalla and Linden, 2012). It is a domain-specific psychological experience influenced by both individual differences and workplace characteristics (McCarthy et al., 2016).

Although WCBA enhances convenience and flexibility in work execution, it inherently increases employees’ job demands. Specifically, WCBA requires employees to continue working outside formal hours—for example, revising work documents during weekends or vacations—which consumes cognitive and emotional resources (Ferguson et al., 2016). At the same time, WCBA requires employees to maintain ongoing attention to work after hours, extending work time and workload while generating worries about task progress, outcomes, or missing important information, thereby inducing anxiety (Eichberger and Zacher, 2021; Derks et al., 2016).

Sustained workplace anxiety further reduces employees’ remaining personal resources, weakening their capacity to fulfill family responsibilities and exacerbating WFC. Prior studies suggest that anxiety and the associated resource depletion hinder employees’ smooth transition between work and family roles, limit their ability to meet family demands, and increase difficulties in handling family-related tasks, thereby aggravating WFC (Bayhan Karapinar et al., 2020). Moreover, employees often struggle to psychologically and behaviorally detach from work after WCBA ends (Bennett et al., 2018), causing work-related behavioral patterns to spill over into personal life, further impeding the effective fulfillment of family responsibilities and ultimately leading to behavioral-level work–family conflict. Combining these arguments, we postulate:

*Hypothesis 2*: Workplace anxiety mediates the relationship between WCBA and WFC.

### The moderating role of PSM

PSM refers to an individual’s psychological tendency to serve others and society for the greater good, it encompasses four dimensions: attraction to public policy-making, commitment to the public interest, compassion, and self-sacrifice (Perry, 1996). Since its introduction, PSM has attracted widespread scholarly attention and become a central topic in public administration and human resource management (Vandenabeele, 2014; Kim et al., 2013). Drawing on the W-HR model, PSM can be viewed as a valuable personal resource that shapes how employees interpret their roles and mobilize other resources (Halbesleben et al., 2014; Bakker and Demerouti, 2017). Prior studies suggest that motivational resources, such as intrinsic and prosocial motivation, often moderate the relationship between job resources and individual outcomes (Ilies et al., 2017; Lin et al., 2017; Bakker et al., 2019). Similarly, research has shown that PSM buffers the negative impact of job demands and fosters resilience in stressful organizational contexts (Bakker, 2015; Borst, 2018; Shim et al., 2017; Ott et al., 2019).

However, the moderating role of PSM in the context of WCBA may be more complex. First, regarding work goal progress, high-PSM employees tend to perceive their work as a mission to advance the public good (Bakker, 2015; Perry et al., 2010). They are more inclined to interpret WCBA as part of their broader service role and to prioritize communal benefits over individual performance goals (Jensen and Vestergaard, 2017). In doing so, they may divert attention and resources away from personal task achievement, thereby weakening the positive effect of WCBA on goal progress. In contrast, employees with low PSM are less guided by public values and more focused on self-interest and concrete task completion (Perry et al., 2010). For them, WCBA is approached more transactionally, without extensive role expansion, which allows its positive impact on goal progress to be more pronounced.

Second, in terms of workplace anxiety, the very traits that make high-PSM employees embrace WCBA also heighten their vulnerability to its psychological costs. Their strong sense of duty, compassion, and self-sacrifice (Perry et al., 2010; Trautmann et al., 2013) causes them to internalize WCBA as a moral obligation. This deep commitment intensifies perceived pressure and role overload (Kamdar et al., 2006), increasing anxiety when demands accumulate or outcomes fall short. Conversely, low-PSM employees, being less emotionally invested in public service values, treat WCBA as a task requirement rather than a moral duty. Their lower level of personal involvement makes them less susceptible to anxiety arising from WCBA. Based on this reasoning, we hypothesize:

*Hypothesis 3a*: PSM negatively moderates the relationship between WCBA and employees’ work goal progress, such that the relationship is weaker for individuals with higher PSM.

*Hypothesis 3b*: PSM positively moderates the relationship between WCBA and employees’ workplace anxiety, such that the relationship is stronger for individuals with higher PSM.

Integrating the above, we expect that Public Service Motivation (PSM) can moderate the influence of WCBA on employees’ work–family conflict through both work goal progress and workplace anxiety. For the resource-gain path via work goal progress, employees with lower PSM are more likely to leverage WCBA to achieve personal work goals, thereby gaining psychological resources that help them manage family responsibilities and reduce WFC. In contrast, employees with higher PSM, who prioritize public service over personal task goals, may be less able to convert WCBA-driven goal progress into personal resource gains, which weakens the beneficial effect on WFC.

For the resource-loss path via workplace anxiety, employees with higher PSM are more likely to experience heightened anxiety when engaging in WCBA due to their strong sense of duty, compassion, and self-sacrifice. This elevated workplace anxiety depletes personal resources, leaving them less able to meet family obligations and thereby increasing WFC. Employees with lower PSM, being less emotionally invested in public service values, are less likely to experience such anxiety, reducing the indirect effect. Accordingly, we hypothesize:

*Hypothesis 4a*: The indirect relationship between WCBA and WFC via work goal progress is moderated by PSM, such that the indirect relationship is weaker for those with higher PSM than for those with lower PSM.

*Hypothesis 4b*: The indirect relationship between WCBA and WFC via workplace anxiety is moderated by PSM, such that the indirect relationship is stronger for those with higher PSM than for those with lower PSM.

## Methods

### Participants and procedures

Data for this study were collected in three waves throughout 2025, involving civil servants from China’s public sector. Against the backdrop of accelerating digital transformation and ongoing government reforms, WCBA has become increasingly prevalent, raising important questions about its effects on work-family relationships. As one of the world’ s fastest-growing major economies, China depends on civil servants as essential actors in policy implementation and public service delivery, thereby placing their wellbeing at the forefront of public sector human resource management. Although WCBA can contribute to improved work efficiency and goal achievement, it may also elevate anxiety levels and intensify WFC. A deeper understanding of these dual mechanisms is crucial for developing targeted policies and managerial practices that enhance administrative performance while protecting civil servants’ wellbeing.

To collect data, we contacted government departments across various public service domains, including administrative services, education, healthcare, and urban management. To reduce the common method variance and any correlation bias, we collected data at three times with an interval of 2 weeks (Podsakoff et al., 2012). Data for WCBA, PSM, and demographic variables were collected at Time 1. At Time 2, data on work goal progress and anxiety were collected. At Time 3, participants completed measures of WFC. Identification codes were used to match responses across the three waves. The research team distributed online survey links via email, on the opening page, participants were provided with an online informed consent form, and confidentiality and anonymity were guaranteed.

In the first wave, a total of 616 questionnaires were received from public servants, and after excluding those who failed attention check questions (e.g., please answer “*strongly disagree*”) and excessively short response times, 512 valid questionnaires were obtained. Similarly, 361 and 240 valid questionnaires were received in the second and third periods, respectively. For the study, 240 participants were finally used, yielding a final effective response rate of 46.8%. The sample includes 93 men (38.7%) and 147 women (61.3%). The average age was 31.2 years (SD = 7.89), and 60.0% of the sample was married. Regarding education level, 29 (12.1%) had a college degree or below, 173 (72.1%) had an undergraduate degree, and 38 (15.8%) had a master’s degree or above. The average number of children and elderly relatives requiring care was 2.33 (SD = 1.35). In terms of administrative rank, 111 (46.3%) were clerks, 59 (24.6%) were staff members, 40 (16.7%) were deputy section chiefs, 23 (9.6%) were section chiefs, and 7 (2.9%) held the rank of deputy division chief or above.

### Measures

#### WCBA

In this study, WCBA was measured using a 3-item scale developed by Ma et al. (2016) in the Chinese context. The scale has been widely validated in subsequent studies, demonstrating good reliability and validity (e.g., Xu et al., 2023). Participants rated each item on a 5-point scale ranging from 1 = *never* to 5 = *very frequently*. A sample item is: “Today, during non-working hours, how often did work-related contacts reach out to me through electronic communication tools because of public matters?” In the present study, the scale showed an internal consistency reliability of 0.78.

#### Work goal progress

Work goal progress was measured using a scale adapted from Koopman et al. (2016). Participants reported their agreement with six statements regarding their perceptions of progress toward work goals since their first survey (1 = *strongly disagree* to 5 = *strongly agree*). In the present study, the items were adapted to the research context. A sample item is: “Using communication tools during non-working hours to handle public service matters has helped me make good progress on my work goals.” The scale demonstrated excellent internal consistency, with a coefficient alpha of 0.93.

#### Workplace anxiety

Workplace anxiety was measured using the eight-item scale developed by McCarthy et al. (2016). Participants rated the extent to which they experienced anxiety related to their work. A sample item is: “I am overwhelmed by thoughts of doing poorly at work.” Each item was rated on a five-point Likert-type scale (1 = *strongly disagree* to 5 = *strongly agree*). The scale demonstrated excellent internal consistency in the present study, with a coefficient alpha of 0.93.

#### PSM

Public service motivation was measured using the short Chinese version of validated by Bao and Li (2016), which originally developed by Kim et al. (2013). Participants rated each of the eight items on a five-point Likert scale ranging from 1 = *strongly disagree* to 5 = *strongly agree*. The scale has demonstrated good psychometric properties, with further validation in Chinese samples (Bao and Li, 2016; Li and Yu, 2025). A sample item is: “For me, it is important to contribute to the public good.” In this study, the internal consistency reliability of this scale was 0.83.

#### WFC

Work–family conflict was measured using the 8-item scale version developed by Carlson et al. (2000), which contains subscales for work interference with family and family interference with work. A sample item is “Due to stress at home, I am often preoccupied with family matters at work.” Each item was rated on a five-point Likert-type scale (1 = *strongly disagree* to 5 = *strongly agree*). In this study, the alpha reliability of the WFC scale was 0.90.

#### Control variables

Following previous research (e.g., Chen et al., 2025), we controlled for demographic characteristics, including gender (1 = female and 2 = male), age, educational level (1 = junior college or below, 2 = bachelor’ s degree, 3 = graduate degree), marital status (1 = single, 2 = married, 3 = divorced), number of elderly relatives and children requiring care, and rank of position (1 = clerk, 2 = staff member, 3 = deputy section chief, 4 = section chief, 5 = deputy division chief, 6 = division chief or above).

## Results

### Common method variance testing

Harman’ s single factor test was used to assess common method bias, the results showed that the variance contribution rate of the first principal component was 23.9%, which was far below the critical threshold of 40%, indicating no serious common method bias in this study. Confirmatory factor analysis (CFA) was conducted using the package ‘lavaan’ in R 4.0.2 (Rosseel, 2012). As shown in Table 1, compared with the other four models, the hypothesized five-factor model (comprising WCBA, work goal progress, workplace anxiety, PSM, and WFC) had a good fit (χ^2^ = 879.95, *df* = 485, *p* < 0.001, CFI = 0.92, TLI = 0.91, SRMR = 0.06, and RMSEA = 0.06) and fit the data better than the single factor model (χ^2^ = 3590.31, *df* = 495, *p* < 0.001, CFI = 0.35, TLI = 0.31, SRMR = 0.22, and RMSEA = 0.16) as well as any other alternative models, evidencing the construct distinctiveness of our study variables. Finally, we followed Jakobsen and Jensen (2015) to run an additional CFA by incorporating an unmeasured latent method construct (ULMC) into the baseline five-factor measurement model to explicitly account for potential common method variance. The results indicated that adding a latent common method variance factor to our theoretical constructs (five-factor model) did not improve the model fit significantly [χ^2^(453) = 817.62, CFI = 0.92, TLI = 0.91, SRMR = 0.06, RMSEA = 0.06], the CFI and TLI improved by less than 0.01, while RMSEA and SRMR remained stable at 0.06. Following the guidelines of Williams and McGonagle (2016), these outcomes indicate that common method variance does not pose a significant threat to the validity of our findings.

**Table 1:** Results of confirmatory factor analyses.

Model	χ^2^	df	χ^2^/df	CFI	TLI	SRMR	RMSEA [90%CI]
Five-factor model^a^	879.95	485	1.81	0.92	0.91	0.06	0.06 [0.05, 0.06]
Four-factor model^b^	1031.36	489	2.11	0.89	0.88	0.08	0.07 [0.06, 0.07]
Three-factor model^c^	2555.54	492	5.19	0.57	0.54	0.19	0.13 [0.13, 0.14]
Two-factor model^d^	2861.92	494	5.79	0.51	0.47	0.20	0.14 [0.14, 0.15]
Single-factor model^e^	3590.31	495	7.25	0.35	0.31	0.22	0.16 [0.16, 0.17]

^a^WCBA, work goal progress, workplace anxiety, PSM, WFC; ^b^WCBA and work goal progress combined; ^c^WCBA, work goal progress, and workplace anxiety combined; ^d^WCBA, work goal progress, and workplace anxiety, and PSM combined; ^e^All factors combined.

### Descriptive statistics

Means, standard deviations, and correlations of the study variables are shown in Table 2. WCBA is positively correlated with work goal progress (*r* = 0.53, *p* < 0.001), workplace anxiety (*r* = 0.20, *p* < 0.01), and PSM (*r* = 0.24, *p* < 0.001), but not significantly correlated with WFC (*r* = −0.02, *p* > 0.05). Work goal progress is positively correlated with PSM (*r* = 0.44, *p* < 0.001), and negatively correlated with WFC (*r* = −0.36, *p* < 0.001). Workplace anxiety is positively correlated with WFC (*r* = 0.42, *p* < 0.001), and negatively correlated with PSM (*r* = −0.24, *p* < 0.001). Additionally, PSM is not significantly correlated with WFC (*r* = −0.11, *p* > 0.05).

**Table 2:** Means, standard deviations, and correlations among study variables.

Variable	1	2	3	4	5	6	7	8	9	10	11
1. Gender	—										
2. Age	0.07	—									
3. Educational level	−0.07	0.00	—								
4. Marital status	0.02	0.65^***^	0.12	—							
5. Care Recipient Numbers	−0.04	0.23^***^	0.12	0.37^***^	—						
6. Rank	0.01	0.50^***^	0.29^***^	0.46^***^	0.16^*^	—					
7. WCBA	0.17^**^	0.08	−0.02	0.12	0.10	0.14^*^	—				
8. Work goal progress	0.16 ^*^	0.08	−0.04	0.20^**^	0.16^*^	0.18^**^	0.53^***^	—			
9. Workplace anxiety	0.02	0.02	−0.04	−0.05	0.07	−0.05	0.20^**^	−0.07	—		
10. PSM	0.10	0.08	0.03	0.21^***^	0.05	0.16^*^	0.24^***^	0.44^***^	−0.24^***^	—	
11. WFC	0.04	−0.06	−0.09	−0.18^**^	−0.03	−0.13	−0.02	−0.36^***^	0.42^***^	−0.11	—
Mean	1.39	31.15	2.04	1.61	2.33	1.99	3.05	3.48	2.84	3.46	2.65
SD	0.49	7.89	0.53	0.50	1.35	1.14	0.76	1.01	0.99	0.63	0.88

*N* = 240; *p < 0.05, **p < 0.01, ***p < 0.001.

### Hypotheses testing

To examine the dual mediating roles of work goal progress and workplace anxiety, we conducted hierarchical regression analyses using Model 4 of the PROCESS macro in SPSS 26.0 (Hayes, 2017). We controlled for employees’ gender, age, educational level, marital status, number of elderly relatives and children requiring care, and rank of position.

As shown in Table 3, WCBA was positively associated with work goal progress (*β* = 0.49, *p* < 0.001), and work goal progress was negatively related to WFC (*β* = −0.40, *p* < 0.001). A bootstrap analysis with 5,000 resamples further indicated that the indirect effect of WCBA on WFC via work goal progress was significant (estimate = −0.27, SE = 0.05, 95% CI = [−0.37, −0.18]). Thus, Hypothesis 1, which predicted a negative indirect relationship between WCBA and WFC through work goal progress, was supported.

**Table 3:** Analyses for the mediating effect of work goal progress and workplace anxiety.

Variable	Work goal progress		Workplace anxiety		WFC			
	*M*_1_		*M*_2_		*M*_3_		*M*_4_	
	*β*	se	*β*	se	*β*	se	*β*	se
Gender	0.08	0.11	−0.02	0.13	0.04	0.12	0.07	0.10
Age	−0.14	0.01	0.13	0.01	0.11	0.01	0.01	0.01
Educational level	−0.09	0.11	−0.01	0.12	−0.05	0.11	−0.08	0.10
Marital status	0.15	0.15	−0.15	0.18	−0.24^**^	0.16	−0.12	0.14
Care recipient numbers	0.08	0.04	0.09	0.05	0.05	0.05	0.05	0.04
Rank	0.13	0.06	0.09	0.07	−0.07	0.06	0.02	0.05
WCBA	0.49^***^	0.07	0.21^**^	0.09	−0.04	0.07	0.11	0.08
Work goal progress							−0.40^***^	0.06
Workplace anxiety							0.36^***^	0.05
*R* ^2^	0.33		0.06		0.05		0.32	
*F*	16.32^***^		2.25^*^		1.69		12.07^***^	

*N* = 240; *p < 0.05, **p < 0.01, ***p < 0.001.

Similarly, WCBA was positively associated with workplace anxiety (*β* = 0.21, *p* < 0.01), and workplace anxiety was positively related to WFC (*β* = 0.36, *p* < 0.001). The bootstrap analysis confirmed a significant indirect effect of WCBA on WFC via workplace anxiety (estimate = 0.11, SE = 0.04, 95% CI = [0.03, 0.18]). Therefore, Hypothesis 2, which predicted a positive indirect relationship between WCBA and WFC through workplace anxiety, was supported.

Hypothesis 3 stated that the moderating role of PSM in the relationships between WCBA and work goal progress (H3a) as well as WCBA and workplace anxiety (H3b). To test the moderation effect of employees’ PSM in this dual impact mechanism, Model 7 in PROCESS was used. As shown in Table 4, the interaction term between WCBA and PSM was significant for both outcomes: it was negatively related to work goal progress (*β* = −0.32, *p* < 0.001) and positively related to workplace anxiety (*β* = 0.38, *p* < 0.01). As presented in Figures 2, 3, simple slope tests confirmed that the positive relationship between WCBA and work goal progress was stronger when PSM was low (*β* = 0.53, *p* < 0.001), but weaker when PSM was high (*β* = 0.20, *p* < 0.01). Similarly, the effect of WCBA on workplace anxiety was nonsignificant when PSM was low (*β* = 0.11, *p* > 0.05), but became significant and stronger when PSM was high (*β* = 0.58, *p* < 0.001). Thus, both Hypothesis 3a and 3b were supported. Figures 2, 3 show the interaction between WCBA and PSM on work goal progress (Figure 2) and workplace anxiety (Figure 3).

**Table 4:** Analyses for the moderation effects of PSM.

Variable	Work goal progress		Workplace anxiety	
	*β*	se	*β*	se
Gender	0.12	0.10	0.01	0.12
Age	−0.01	0.01	0.01	0.01
Educational level	−0.14	0.10	−0.03	0.12
Marital status	0.20	0.14	−0.20	0.17
Care recipient numbers	0.06	0.04	0.07	0.05
Rank	0.07	0.05	−0.05	0.06
WCBA	0.57^***^	0.07	0.35^***^	0.08
PSM	0.54^***^	0.08	−0.48^***^	0.10
WCBA*PSM	−0.32^***^	0.10	0.38^**^	0.13
*R* ^2^	0.35		0.16	
*F*	18.97^***^		5.03^***^	

*N* = 240; *p < 0.05, **p < 0.01, ***p < 0.001.

**Figure 2:**
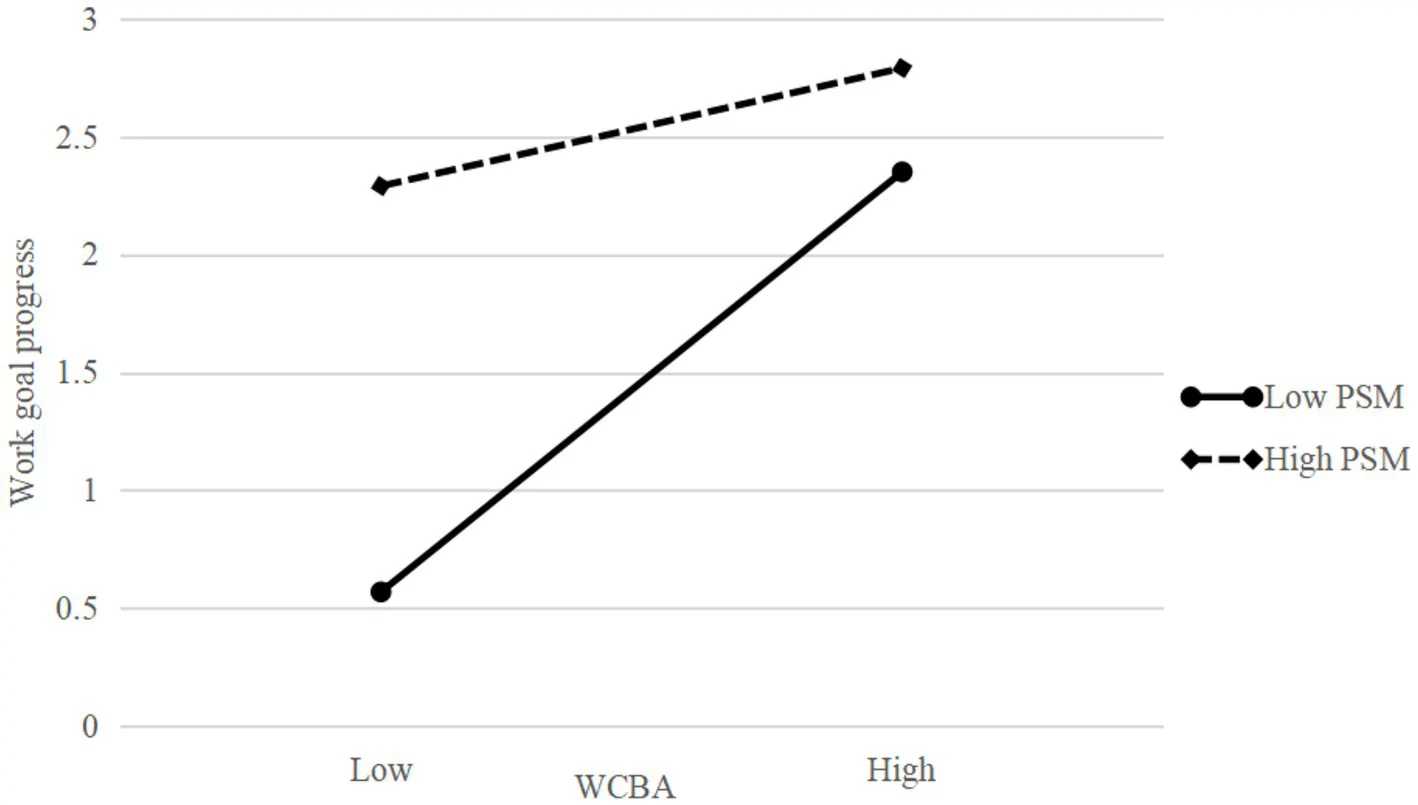
Moderating effect of PSM on the relationship between WCBA and work goal progress.

**Figure 3:**
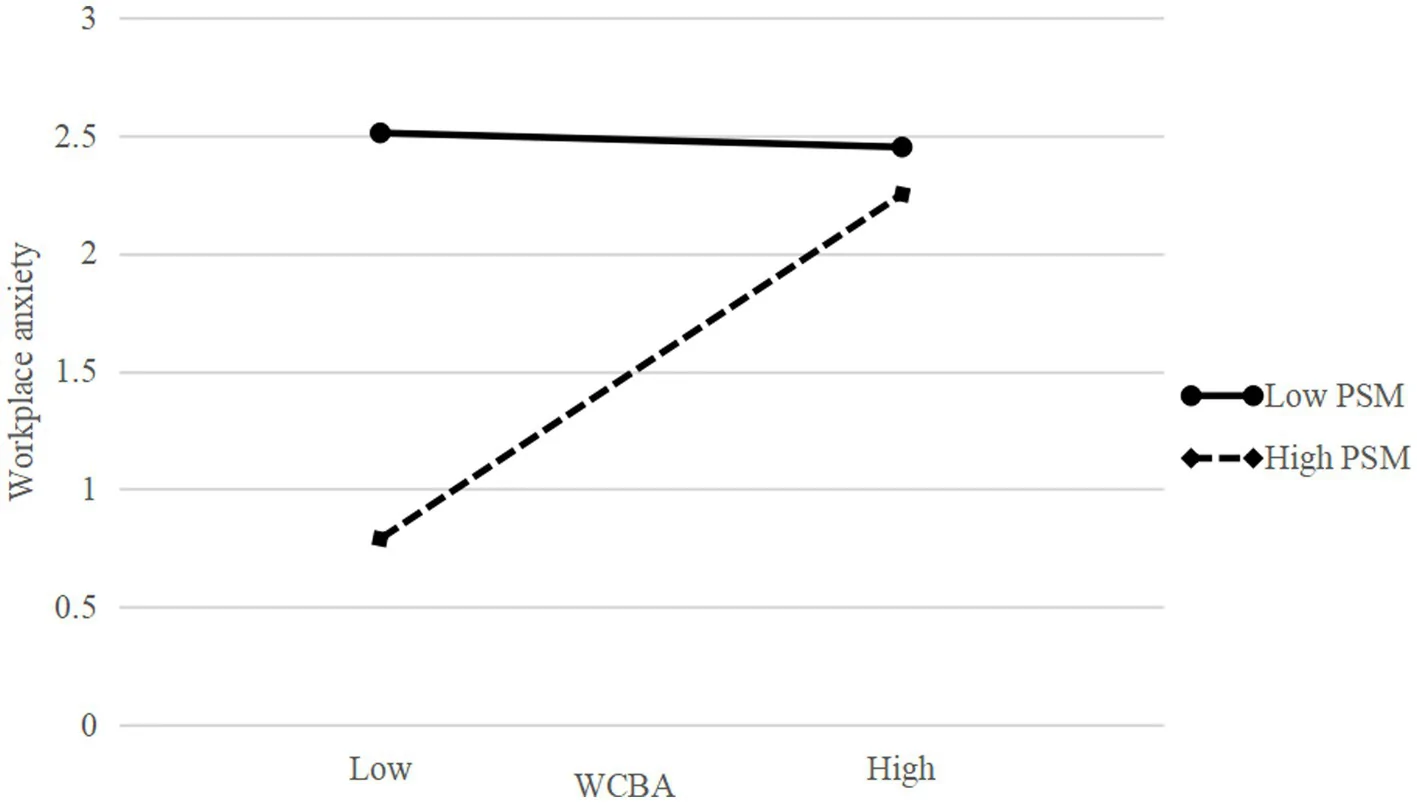
Moderating effect of PSM on the relationship between WCBA and workplace anxiety.

We further tested whether the indirect relationships between WCBA and WFC via work goal progress (H4a) and workplace anxiety (H4b) were moderated by PSM. Table 3 presents the results of the conditional indirect relationships. Further bootstrap analysis with 5,000 resamples revealed that the indirect effect of WCBA on WFC through goal progress was significant at all levels of PSM, the conditional indirect effects is stronger when PSM is low (–1 SD, *effect* = −0.25; SE = 0.05; 95% CI = [−0.40, −0.12]) compared to when PSM is high (+1 SD, *effect* = −0.10; SE = 0.04; 95% CI = [−0.20, −0.05]). Moreover, the index of moderated mediation was significant (index = 0.18, SE = 0.06, 95% CI [0.07, 0.29]), the difference between these two mediating effects is significant (difference = 0.17, SE = 0.06; 95% CI = [0.05, 0.29]). Indicating that PSM significantly moderated the indirect effect of WCBA on WFC via goal progress.

Bootstrap analysis (5,000 resamples) further showed that the indirect effect of WCBA on WFC via anxiety differed across levels of PSM. The conditional indirect effects is weaker when traditionality is low (–1 SD, *effect* = 0.04; SE = 0.04; 95% CI = [−0.06, 0.13]) compared with when traditionality is high (+1 SD, *effect* = 0.22; SE = 0.05; 95% CI = [0.13, 0.33]). The index of moderated mediation was significant (index = 0.14, SE = 0.05, 95% CI [0.05, 0.26]), the difference between these two mediating effects is significant (difference = 0.18, SE = 0.06; 95% CI = [0.06, 0.33]). Suggesting that PSM significantly moderated the indirect effect of WCBA on WFC via anxiety. Thus, Hypothesis 4a and 4b were supported.

## Discussion

While most previous studies have examined the effects of WCBA on corporate employees and produced inconsistent findings regarding family-related outcomes, its implications for civil servants remain largely unexplored. To address this gap, the present study investigates the paradoxical effects of WCBA on civil servants’ WFC. Drawing on the W-HR model, the findings reveal a dual pathway: on the one hand, WCBA facilitates work goal progress, which generates valuable psychological resources that spill over positively into the family domain, thereby alleviating WFC; on the other hand, WCBA induces workplace anxiety, which consumes employees’ cognitive and emotional resources and ultimately exacerbates WFC. Furthermore, PSM acts as a boundary condition, with the gain path of WCBA through work goal progress being weaker for employees with high PSM, while the loss path through workplace anxiety is stronger for those with high PSM. These findings contribute to a more nuanced understanding of the double-edged nature of WCBA and the complex role of PSM in shaping the work–family dynamics of Chinese civil servants.

### Theoretical implications

This study holds significant theoretical implications. First, it contributes to the existing literature on WCBA and WFC, particularly within the context of Chinese public organizations. Most prior research has primarily focused on the effects of WCBA on work-related outcomes, such as task performance, job satisfaction, work engagement, and organizational commitment (e.g., Ferguson et al., 2016; Ren et al., 2023), while its impact on non-work domains, especially family life, remains inconsistent (Gadeyne et al., 2023; Golden, 2012; van Zoonen et al., 2020). More importantly, previous studies have largely examined employees in corporate settings, leaving the implications of WCBA for civil servants underexplored (Liu and Shi, 2024; Wang and Li, 2023). The rapid development of ICT has profoundly transformed work practices in the public sector, making the handling of work-related tasks after hours increasingly routine (Dawes, 2008; Roman et al., 2019; Wang and Li, 2023). In particular, Chinese public organizations, which emphasize collectivism and the public service ethos, and prioritize public interests over personal needs (Chen et al., 2025), represent a context where the effects of WCBA on civil servants’ personal and family well-being are particularly relevant but understudied. Therefore, this study extends existing research by exploring both the beneficial and adverse impacts of WCBA on WFC among Chinese civil servants, providing a more balanced understanding of how WCBA affects employees’ non-work domains.

Second, this study advances theoretical understanding by applying the W-HR Model to integrate both the resource-loss and resource-gain perspectives in examining the effects of WCBA on civil servants’ WFC. Although previous studies have begun to acknowledge the potential impact of WCBA on family outcomes, the findings remain inconsistent, and the underlying mechanisms and boundary conditions are yet to be fully explored (Gadeyne et al., 2023; Golden, 2012; van Zoonen et al., 2020). Drawing on the W-HR Model, this study integrates dual theoretical perspectives to systematically investigate the impact of WCBA on civil servants’ WFC, with work goal progress representing the resource-gain path and workplace anxiety representing the resource-loss path. On one hand, WCBA facilitates work goal progress by enabling employees to flexibly allocate time and effort, access additional job resources, and improve task completion, thereby generating positive psychological resources that spill over into the family domain and alleviate WFC. On the other hand, WCBA extends working hours, increases cognitive and emotional demands, and triggers workplace anxiety, depleting personal resources and exacerbating WFC. By simultaneously examining both resource-gain and resource-loss paths, this study reveals the dual-edged effect of WCBA on Chinese civil servants’ WFC and provides a more comprehensive theoretical explanation of how after-hours work connectivity influences non-work outcomes among civil servants.

Third, by examining the moderating effect of PSM, this study reveals the boundary conditions under which WCBA generates either gains or losses in WFC. Along the resource-gain pathway, employees with high PSM tend to view WCBA as part of their public duty, prioritizing collective interests over personal goals. This orientation diverts attention from individual work progress, thereby weakening WCBA’s positive effect on WFC reduction. In contrast, low-PSM employees, being less driven by public values, focus more on personal tasks and are better able to leverage WCBA to facilitate goal attainment and mitigate WFC. Along the resource-loss pathway, the strong sense of duty, compassion, and self-sacrifice characteristic of high-PSM employees leads them to internalize WCBA as a moral obligation. When work demands extend beyond regular hours, this heightened sense of mission intensifies workplace anxiety, drains personal resources, and aggravates WFC. Conversely, low-PSM employees treat WCBA more transactionally, making them less prone to anxiety and less affected by its negative impact. These findings align with prior research underscoring the moderating role of personal resources in shaping the work–family interface (Ilies et al., 2017; Lin et al., 2017; Bakker et al., 2019).

Moreover, this study enriches the theoretical understanding of PSM by highlighting both its bright and dark sides. While traditionally regarded as a positive personal resource—enhancing job satisfaction, commitment, performance, and civic engagement (Harari et al., 2016; Qi and Wang, 2018; Miao et al., 2018)—recent studies have also pointed to its potential downsides, including stress, burnout, absenteeism, and turnover intentions (Giauque et al., 2012; Schott and Ritz, 2018; Chen et al., 2025). Our findings show that in the absence of organizational support, high PSM may paradoxically function as a resource-depleting mechanism: employees set unrealistically high standards, suffer stress when goals are unattainable, and overinvest personal resources in public welfare, ultimately compromising their well-being (Giauque et al., 2012; Gould-Williams et al., 2015; Liu et al., 2015; Li and Chen, 2023). By uncovering the latent dark side of PSM within the “always-on” WCBA context, this study demonstrates that while PSM is generally regarded as a valuable motivational resource, its effects may also involve unintended costs under certain conditions. This finding advances a more dialectical and nuanced understanding of PSM theory.

### Managerial implications

First, as WCBA operates through both resource-gain and resource-loss pathways, managers should design its use with a balanced perspective. Digital connectivity can support work goal achievement and generate psychological resources, but excessive or poorly regulated connectivity may heighten workplace anxiety. Therefore, managers need to clearly define expectations regarding after-hours work, specifying when and how employees are expected to respond, and establish fair rotation systems for on-call duties.

Second, managers should adopt differentiated management strategies to account for employees’ varying levels of PSM. High-PSM employees are more likely to interpret WCBA as a moral obligation, which can heighten their anxiety and deplete resources. To mitigate this risk, organizations should provide targeted emotional support, regular one-on-one mental check-ins, and structured off-work recovery opportunities. Supervisors can track the effectiveness of such interventions via quarterly employee well-being surveys and periodic stress assessments. In contrast, low-PSM employees may not automatically view WCBA as duty-driven. For them, managers can highlight the instrumental value of WCBA, such as improving efficiency and enabling better work–family balance, so that it functions as a motivating rather than draining mechanism.

Third, organizations should foster a work culture that emphasizes sustainable engagement rather than constant availability. Although PSM is generally valued as a motivational asset, this study highlights its potential to backfire under an always-connected work regime. Managers should thus avoid reinforcing over-commitment as a cultural norm and instead promote clear boundaries, realistic performance expectations, and healthy recovery practices. Leadership communication should encourage employees to disconnect during personal time and model responsible digital practices.

Finally, managers should complement WCBA practices with broader institutional support to buffer its negative spillover into family life. This includes introducing tangible family-friendly policies such as flexible scheduling, childcare subsidies, or compensatory leave. When rolling out these policies, organizations must acknowledge and address common implementation barriers, including heavy public sector workload pressure, rigid hierarchical culture, and ingrained norms of after-hours responsiveness that create cultural resistance to boundary-setting. Organizations can mitigate such resistance through top-down leadership advocacy and gradual policy pilot trials. In addition, firms can offer long-term psychological counseling and peer-support programs. By embedding WCBA within a broader support system, public organizations can reduce workplace anxiety, sustain employees’ psychological resources, and ultimately strengthen both organizational performance and employee well-being.

### Limitations and future directions

While this study offers important insights into the dual effects of WCBA on civil servants’ WFC, several limitations suggest directions for future research. First, although the time-lagged survey design helps mitigate common method bias, it may not fully capture the dynamic and evolving nature of WCBA, workplace anxiety, and work goal progress over time. Future studies could employ experimental or longitudinal designs, for example, use experience sampling methods (ESM) or diary designs, to better capture intra-day dynamic causal processes.

Second, this study has several interrelated limitations regarding variables and theoretical framework. We only measured general work–family conflict rather than specific outcomes such as marital satisfaction and parent–child interactions, limiting our understanding of differentiated spillover effects of WCBA (French et al., 2018). Meanwhile, we merely adopted work goal progress and workplace anxiety as mediators, omitting personal resilience, social support and recovery experiences emphasized by the W-HR Model, which may oversimplify work-family resource dynamics. Besides, key organizational factors including organizational culture, leadership style and digital policies were not examined. Future research can enrich family-related outcome indicators, expand potential mediating pathways, and incorporate organizational contextual variables to construct a more comprehensive model.

Finally, this study was conducted within the context of Chinese public organizations, where collectivism and public service motivation play a central role. As Chen et al. (2025) and Wang and Li (2023) noted, work-life boundaries and work motivation differ across cultures and industries, so our findings have limited generalizability. The results may not extend to corporate employees or civil servants in less collectivist regions. Future research could test our model across different occupations and cultural backgrounds, including variations in PSM, collectivist values, and organizational norms, shape the dual pathways of WCBA, thereby enriching our understanding of work–family processes in different cultural and institutional settings.

## Data Availability

The original contributions presented in the study are included in the article/supplementary material, further inquiries can be directed to the corresponding author/s.
